# GMDS knockdown impairs cell proliferation and survival in human lung adenocarcinoma

**DOI:** 10.1186/s12885-018-4524-1

**Published:** 2018-05-29

**Authors:** Xing Wei, Kun Zhang, Haifeng Qin, Jinlong Zhu, Qiaoxi Qin, Yang Yu, Hong Wang

**Affiliations:** 1 Department of Lung Cancer, The Affiliated Hospital of Military Medical Sciences, The 307th Hospital of Chinese People’s Liberation Army, Beijing, 100071 China; 2 Outpatient Department, Southern Theatre Command of People’s Liberation Army, Guangzhou, 510080 Guangdong China

**Keywords:** Lung adenocarcinoma, GMDS, Cell proliferation, Survival, Tumorigenesis, Molecular network

## Abstract

**Background:**

Lung adenocarcinoma is the most common type of lung cancer and one of the most lethal and prevalent cancers. Aberrant glycosylation was common and essential in tumorigenesis, with fucosylation as one of the most common types disrupted in cancers. However, it is still unknown whether genes involved in fucosylation are important for lung adenocarcinoma development and process.

**Methods:**

GMDS is involved in cellular fucosylation. Here we examined GMDS expression level at both mRNA and protein level in lung adenocarcinoma. The impact of GMDS knockdown on lung adenocarcinoma in vitro and in vivo was investigated. Transcriptome changes with GMDS knockdown in lung adenocarcinoma cells were also examined to provide insights into related molecular mechanisms.

**Results:**

GMDS expression is significantly upregulated in lung adenocarcinoma at both mRNA and protein levels. Lentivirus-mediated shRNA strategy inhibited GMDS expression efficiently in human lung adenocarcinoma cells A549 and H1299, and GMDS knockdown impaired cell proliferation, colony formation ability, induced cell cycle arrest, and apoptosis in both cell lines. Furthermore, GMDS knockdown inhibited tumorigenesis in a xenograft mice model of lung adenocarcinoma. Microarray analysis explored the GMDS-mediated molecular network and revealed that the CASP8-CDKN1A axis might be critical for lung adenocarcinoma development.

**Conclusions:**

These findings suggest that GMDS upregulation is critical for cell proliferation and survival in human lung adenocarcinoma and might serve as a potential biomarker for lung adenocarcinoma diagnosis and treatment.

**Electronic supplementary material:**

The online version of this article (10.1186/s12885-018-4524-1) contains supplementary material, which is available to authorized users.

## Background

Lung cancer is one of the most lethal and prevalent human cancers, and its five-year survival rate is currently less than 20% [1, 2]. Lung cancer can be classified into non-small cell lung cancer (NSCLC) and small cell lung cancer (SCLC), with NSCLC accounting for approximately 85% of all lung cancers [3]. The most common subtype of NSCLC is lung adenocarcinoma, which accounts for approximately 40% of all lung cancers and leads to more than 500,000 deaths each year [4]. In recent years, with the development of high-throughput sequencing technologies, molecular profiling of lung adenocarcinoma has been described and multiple oncogenic mutations have been identified, which might serve as markers for diagnosis and targeted therapies [5–7]. However, these results were far from sufficient, and the diagnosis of many lung adenocarcinoma patients remains poor, as most lung adenocarcinoma patients are already at advanced or metastatic stages when first diagnosed owing to the lack of suitable diagnostic markers [8, 9]. Thus, there is still an urgent need to explore the molecular pathways underlying lung adenocarcinoma tumorigenesis and progression for insights into the identification of appropriate markers, which will guide the development of novel diagnostic strategies and targeted therapies in the future.

Glycosylation, a process of attaching saccharides to proteins, saccharides, or lipids, is an important post-translational modification involved in variant biological physiological functions [10]. Glycosylation defects have been linked to many pathophysiological processes, including inflammation, tumorigenesis, and cancer metastasis [11, 12]. Glycosylation can be divided into approximately 10 different kinds of oligosaccharide modifications according to the oligosaccharide structure, with fucosylation as one of the most common types deregulated in cancers [13]. In the process of cellular fucosylation, GDP-fucose serves as the essential substrate, and its synthesis was driven by GDP-mannose-4,6-dehydratase (GMDS) and GDP-4-keto-6-deoxymannose-3,5-epimerase-4-reductase (FX) [14]. As fucosylation changes have been linked to different cancers, and FX expression changes were also observed in liver cancer cells, it is possible that GMDS might be involved in cancer development [15]. GMDS mutations have been shown to be positively related to colorectal cancer metastasis, and GMDS deficiency accounts for tumor escape and resistance to cellular apoptosis in colon cancer cells [16–18].

Here, we first analyzed gene expression profiling in 57 paired lung adenocarcinoma cases from The Cancer Genome Atlas (TCGA) database and found that GMDS expression was significantly upregulated in lung adenocarcinoma tissues as compared to adjacent normal tissues. GMDS upregulation was further confirmed in specimens from lung adenocarcinoma patients using immunohistochemistry. Then, two lung adenocarcinoma cell lines A549 and H1299 cells were chosen for further functional analysis of GMDS. Lentiviral–mediated short hairpin RNA (shRNA) specifically targeting human GMDS was employed to inhibit GMDS expression in lung adenocarcinoma cells, and knockdown efficiency was confirmed at both mRNA and protein level by quantitative real-time polymerase chain reaction (qPCR) and western blot. GMDS knockdown in A549 cells and H1299 cells impaired cell proliferation and colony formation ability and induced cell cycle arrest and apoptosis. Furthermore, nude mice tumorigenesis assays showed that GMDS knockdown inhibited tumor growth in vivo. To explore the molecular mechanisms underlying GMDS-associated cellular processes, a microarray assay was used to investigate gene expression changes induced by GMDS knockdown, and potential targets of GMDS were further validated by western blot. Taken together, our study revealed that GMDS was upregulated in lung adenocarcinoma tissues, which might serve as a biomarker for lung adenocarcinoma. We provided confidential evidence that GMDS might serve as a tumor-promoting factor in lung adenocarcinoma, which is in contrast to the reported anti-tumor functions in colon cancers. It is necessary to further determine GMDS functions in other cancer types to provide a comprehensive profile for GMDS in tumorigenesis and progression.

## Methods

### Tissue microarray and immunohistochemistry (IHC) for GMDS

Tissue microarray (HLug-Ade150CS-01) containing 75 pairs of formalin-fixed, Paraffin-embedded (FFPE) human lung adenocarcinoma and adjacent normal sample were obtained from Outdo Biotech Company (Shanghai, China). Detailed information about clinical parameters for these patients was summarized in Table 1. Immunohistochemistry for GMDS protein expression status was carried out in tissue microarray as follows: antigen unmasking was performed using 10 mM sodium citrate buffer at 80 °C for 20 min. Tissue sections were then blocked in blocking buffer for 60 min at 25 °C and further treated with GMDS antibody (Novus, NBP1–33424,1:100) at 4 °C for 12~ 16 h. After washing 2 times for 30 min using PBS buffer, immunohistochemistry staining was performed using biotin-labeled secondary antibody, diaminobenzidine and counterstained with hematoxylin. Negative controls were performed without primary antibody against GMDS. GMDS expression status was determined by two pathologists blindly and independently with the following standards: intensity score was distinguished as score 3, strong positive signal, 2, moderate positive signal, 1, weak positive signal and score 0, no staining signal; while positive rate was scored as 0, negative; 1, 1–25%; 2, 26–50%; 3, 51–75%; 4, 76–100%. The final immunoreactions score was quantified as GMDS immunoreactivity = intensity score*positive rate. Samples with the final scores ≤2 were defined as GMDS low status while others were considered as GMDS high status. Mann-Whitney U method was used for statistical analysis.

**Table 1:** Relationship between GMDS expression and clinical pathological parameters (cases,%)by IHC staining

Clinical pathological parameters	Case number	HSDL2 expression status		*P value*
		low	high	
Ages(year)				
≤ 65	111	53	58	0.080
> 65	54	18	36	
Gender				
Male	88	39	49	0.776
Female	76	32	44	
Tumor diameter(cm)				
≤ 5	137	62	75	0.203
> 5	28	9	19	
Grades				
I/II	126	54	72	0.936
III	39	17	22	
T staging				
T1/T2	128	55	73	0.653
T3/T4	37	16	21	
N migration				
Nx/N0	99	42	57	0.653
N1/N2/N3	63	29	34	
M metastasis				
M0	159	70	89	0.287
M1	5	1	4	
TNM staging				
TNM1/2	112	51	61	0.580
TNM3/4	49	20	29	

### Expression profiles and clinical outcome analysis

Fifty seven paired lung adenocarcinoma samples with RNAseq data from TCGA was used for gene profiling analysis. Expression profile of GSE31210 was downloaded and predictive value of GMDS with relapse-free survival was analyzed with Pan Cancer Prognostics Database PROGgeneV2.

### Cell culture

Two human lung adenocarcinoma cell lines, namely A549 cells and H1299 cells, were purchased from Cell Bank of the Chinese Academy of Sciences (Shanghai, China). A549 cells and H1299 cells were cultured in RPMI1640 medium containing 10% FBS and 1% antibiotics. These cell lines were cultured at 37 °C in a 5% CO_2_ incubator.

### Design of GMDS shRNA and lentivirus production

Short-hairpin RNA (shRNA) targeting human GMDS gene (sequence: 5’-CGTGAGGCGTATAATCTCTTT-3′) was designed and oligonucleotides were synthesized by GeneChem (Shanghai, China). Then oligonucleotides were then annealed and inserted into a lentiviral vector pGCSIL-GFP with AgeI and EcoRI (both from NEB, Ipswich, MA, USA). Lentivirus expressing GMDS shRNA was produced as previously described [19]. The Lentivector Expression System (GeneChem, Shanghai, China) was used for lentivirus expressing GMDS shRNA (GMDS-shRNA) or scrambled shRNA (Scr-shRNA, negative control, sequence: 5’-TTCTCCGAACGTGTCACGT-3′).

### GMDS expression analysis using TCGA database

Fifty seven paired lung adenocarcinoma samples which have been analyzed by RNA sequencing in TCGA database were selected and transcriptome information was downloaded. Gene expression profiling for these paired samples were analyzed by Log_2_ (lung adenocarcinoma tissues/adjacent normal tissues).

### Infections of human lung adenocarcinoma cells with lentivirus

Human lung adenocarcinoma cell line A549 cells and H1299 cells were used for GMDS studies. In brief, cells were plated in 6-, 12- or 24-well plates according to experiments and incubated in a 5% CO_2_ incubator at 37 °C to achieve desired density. Then, lentivirus expressing either GMDS- or Scr-shRNA was added to the target plate (MOI = 5 and 5 ul lentivirus was used per well for 6-well plate). After culturing for another 2–5 days, cellular infection efficiency was examined according to the percentage of GFP-positive cells observed using a fluorescence microscope.

### RNA extraction, cDNA synthesis and real-time quantitative PCR

A549 cells and H1299 cells infected with lentivirus expressing either Scr-shRNA or GMDS-shRNA were cultured for 5 days and then harvested for total RNA extraction, reverse transcription and quantitative PCR. Briefly, Trizol reagent (Invitrogen, Carlsbad, CA, USA) was used for RNA purification. RNA was then quantified using NanoDrop (Thermo, Rockford, IL, MA, USA) and the first-strand cDNA was produced using Oligo dT primers (Sangon, Shanghai, China) and M-MLV reverse transcriptase (Promega, Madison, Wisconsin, USA). The expression of GMDS and other genes was quantified with SYBR mixture (Takara Biotechnology, Dalian, China) on a Real-Time PCR machine TP800 (Takara Biotechnology, Dalian, China). Beacon designer 2 was used for primer design and these primers were synthesized by GeneChem (Shanghai, China). Lists of primers were as follows:

GAPDH forward: 5’-TGACTTCAACAGCGACACCCA-3′;

GAPDH reverse: 5’-CACCCTGTTGCTGTAGCCAAA-3′;

GMDS forward: 5′- TTTAATACGGGTCGAATTGAGCA-3′;

GMDS reverse: 5′- TGAGATCGCCATAGTGCAACT-3′;

SKA1 forward: 5′- ATGAAGAAACGAAGGATACCAAAG-3′;

SKA1 reverse: 5′- CCTCGGACCTCTGATAGCC-3′;

VEGFA forward: 5’-GCTTACTCTCACCTGCTTCTG-3′;

VEGFA reverse: 5′- GGCTGCTTCTTCCAACAATG-3′;

DDIT3 forward: 5′- CTTCTCTGGCTTGGCTGACTGA-3′;

DDIT3 reverse: 5′- TGACTGGAATCTGGAGAGTGAGG-3′;

MAD2L1 forward: 5′- GAGTCGGGACCACAGTTTAT-3′;

MAD2L1 reverse: 5′- TTTTGTAGGCCACCATGCTA-3′;

MAP3K7 forward: 5′- CCGGTGAGATGATCGAAGCC-3′;

MAP3K7 reverse: 5′- GCCGAAGCTCTACAATAAACGC-3′;

CDKN1A forward: 5′- CTGTCACTGTCTTGTACCCTTGT-3′;

CDKN1A reverse: 5′- AAATCTGTCATGCTGGTCTGC-3′;

FAS forward: 5′- CTTCTTTTGCCAATTCCAC-3′;

FAS reverse: 5′- CAGATAAATTTATTGCCACTG-3′;

CASP8 forward: 5′- TTTCTGCCTACAGGGTCATGC-3′;

CASP8 reverse: 5′- TGTCCAACTTTCCTTCTCCCA-3′;

JUN forward: 5′- CGCCAAGAACTCGGACCTC-3′;

JUN reverse: 5’-CCTCCTGCTCATCTGTCACG-3′;

Relative gene expression was normalized to GAPDH, and data analysis was performed using the delta-delta CT method.

### Immunoblotting

A549 cells and H1299 cells infected with lentivirus expressing either Scr-shRNA or GMDS-shRNA were cultured for 2 days for protein isolation. In brief, cells were washed with PBS buffer and harvested with lysis buffer (100 mM Tris-HCl, pH = 7.4 l 0.15 M NaCl; 5 mM EDTA, pH = 8.0; 1% Triton X100; 5 mM DTT; 0.1 mM PMSF) to extract total proteins which were quantified with BCA Protein Assay Kit (Pierce, Rockford, IL, USA). To perform western blot analysis, 20 μg protein samples were mixed with loading buffer. Then SDS-PAGE electrophoresis and subsequent PVDF transmembrane were performed (Amersham Biosciences, Pollards Wood, UK). Membrane was blocked with 5% milk dissolved in TBST buffer for 1 h and then incubated with primary antibodies overnight at 4 °C. Primary antibodies used here were as follows: Rabbit anti-GMDS, Novus Biological, NBP1–33424 (1:500); Rabbit anti-CDKN1A, Abcam, ab7960 (1:500); Mouse anti-DDIT3, Abcam, ab11419 (1:1000); Rabbit anti-FAS, Abcam, ab82419 (1:1000); Rabbit anti-JUN, Abcam, ab32137 (1:1000); Rabbit anti-VEGFA, Abcam, ab183100 (1:500); mouse anti-Flag, Sigma, F1804 (1:1000); mouse anti-GAPDH, Santa-Cruz, sc-32,233 (1:2000). After washing with TBST buffer for three times, specific HRP conjugated secondary antibodies from Santa Cruz were added and immunoactivity was detected with ECL-Plus kit (Amersham Biosciences, Pollards Wood, UK).

### Cell proliferation analysis using Cellomics ArrayScan VTI

Lentivirus expressing GMDS- or Scr-shRNA was added used here for cell infection. After culturing for another 2 days, cells were re-seeded in 96-well plates with 2000 cells/well in triplicate. After incubating for another 24 h, cell growth was examined with Cellomics ArrayScan VTI (Thermo, Rockford, IL, MA, USA) once a day for 5 days to produce cell growth curves.

### MTT assay

Lentiviral-infected A549 cells and H1299 cells were cultured to reach logarithmic phase. Cells were then collected for cell number counting using a hemocytometer. Then cells were re-seeded into 96-well plates with 2000 cells/well in triplicate for further culturing. Cells were treated with 20 μL of MTT solution (5 mg/mL) per well and incubated for 4 h. Then culture medium replaced with150 μL of DMSO for formazan dissolving. After incubating for 5–10 min, the absorbance at 490/570 nm was examined using a microplate reader.

### Colony formation assay

As described previously [20], Lentiviral-infected A549 cells and H1299 cells were cultured for 2 days and harvested in the logarithmic phase. After cell counting, cells were re-seeded into six-well plates with 800 cells/well in triplicate and cultured at 37 °C for 2 weeks. Cells were then fixed using paraformaldehyde for 30–60 min and stained with GIEMSA for 20 min. After washing with ddH_2_O thoroughly, cell plate imaging was obtained with micropublisher 3.3RTV (Olympus) for the quantification of cell colonies.

### Cell cycle analysis with flow cytometry

Cell cycle analysis was done as previously reported [21]. Lentiviral-infected A549 cells and H1299 cells were cultured for 48 or 72 h. Cells were harvested and fixed with cold 70% ethanol for about 1 h. After PBS washing, cells were incubated with PI buffer (40 × PI stock (2 mg/ml), 100 × RNase stock (10 mg/ml) and 1 × PBS buffer at a dilution of 25:10:1000). FACS Calibur (Becton-Dickinson, San Jose, CA, USA) was then used to analyze cell cycle status. More than 1 × 10^6^ cells per sample were used for each experiment, and experiments were performed in triplicate.

### Annexin V-APC assay for cell apoptosis analysis

Annexin V-APC apoptosis detection kit (eBioscience, San Diego, CA, USA) was used here. Initially, Lentiviral-infected A549 cells and H1299 cells were cultured for 96 h. Cells were subsequently harvested to produce cell suspensions containing 1 × 10^6^–1 × 10^7^/ml cells using staining buffer. Then, 5 μl annexin V-APC was added into 100 μl cell suspensions and incubated at 25 °C for 10–15 min. FACS Calibur (Becton-Dickinson, San Jose, CA, USA) was used for cell apoptotic analysis.

### Caspase3/7 activity analysis

Caspase-Glo® 3/7 Assay (Promega, Madison, Wisconsin, USA) was used for Caspase3/7 activity analysis according to the manufacture’s manual. In brief, A549 cells and H1299 cells infected with lentivirus expressing either Scr-shRNA or GMDS-shRNA were cultured for 3–5 days, After harvesting and cell counting using a haemocytometer, cells were seeded into 96-well plates at a density of 1 × 10^4^ cells/well. Then 100 μl Caspase-Glo reaction buffer were added into cells per well and cell plate were shaken constantly at 300–500 rpm for 30 min. Then signals were quantified after cells were incubated at room time for 1–2 h.

### Tumorigenicity in nude mice

Nude mice experiments were approved by the Institutional Animal Care and Use committee and all nude mice were feeded strictly following the institution guidelines. A lung adenocarcinoma xenograft model was established as follows: Lentiviral-infected H1299 cells were cultured to reach logarithmic phase. After harvesting and cell counting, cells suspensions of 2 × 10^7^ cells/ml were prepared with PBS buffer. Then approximately 4 × 10^6^ cells were injected into nude mice subcutaneously. Then mice were divided into two groups: control group were injected with H1299 cells infected with Scr-shRNA lentivirus and knockdown group injected with H1299 cells infected with GMDS-shRNA lentivirus. Tumour diameter in these nude mice was examined every other day from the 10th day for 7 times two times a week. Tumor weight was determined at 29th day after killing nude mice.

### Microarray analysis for gene expression profiles in A549 cells

A549 cells infected with lentivirus expressing either Scr-shRNA (*n* = 3) or GMDS-shRNA (*n* = 3) were cultured and then total RNA was extracted using Trizol reagents. NanoDrop 2000 and Agilent Bioanalyzer 2100 were used for RNA examination. For gene expression analysis, Affymetrix human GeneChip primeview was used according to manuals as described previously [22]. In brief, GeneChip 3’ IVT Expression Kit was used for first-strand complementary DNA synthesis, double-stranded DNA template conversion, in vitro transcription for aRNA synthesis and labelling. Microarray hybridization, washing and staining were done using GeneChip Hybridization Wash and Stain Kit. GeneChip Scanner 3000 was used for array scanning to produce raw data. Gene expression profiles in A549 cells infected with lentivirus expressing Scr-shRNA (*n* = 3) or GMDS-shRNA (*n* = 3) were analyzed to identify differentially expressed genes based on the following criteria: *P* value < 0.05 and absolute fold change > 2. Pathway enrichment and gene network analysis were done based on Ingenuity Pathway Analysis (IPA). Microarray data is accessible through GEO series accession number GSE104123.

#### Statistical analysis

GraphPad Prism 6 was used for data analysis, and all experiments were done in triplicate. Data are shown as the mean ± SEM of three independent experiments. Student’s two-tailed t-test was chosen for statistical analysis and *P* < 0.05 was considered statistically significant. For the analysis of the difference in GMDS expression between lung adenocarcinoma samples and adjacent normal samples, Fisher’s exact test was used.

## Results

### Upregulation of GMDS expression in human lung adenocarcinoma

**Fig. 1:**
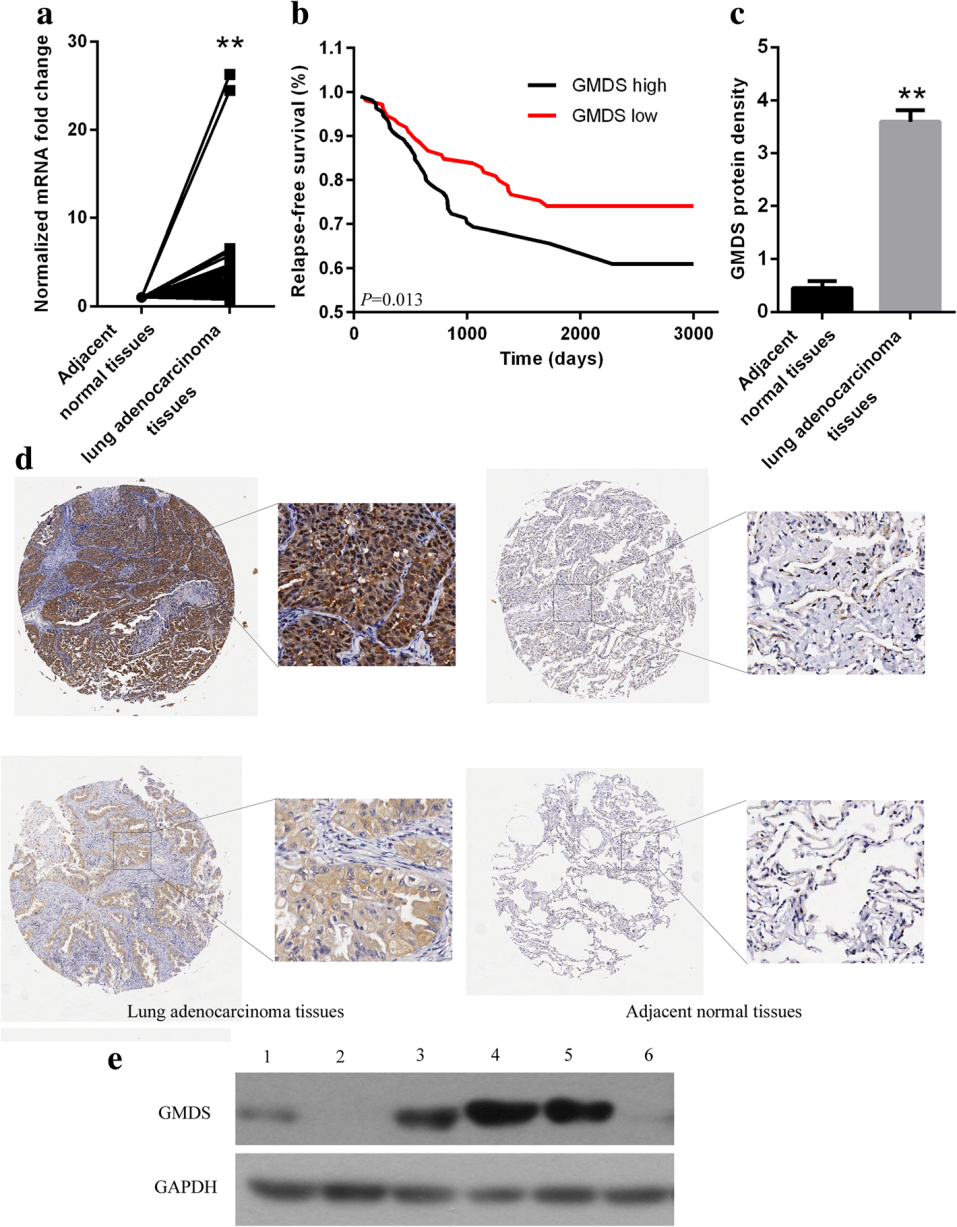
GMDS expression levels in human lung adenocarcinoma tissues and cells. **a** GMDS mRNA level in human lung adenocarcinoma tissues and adjacent normal tissues. Fifty seven paired lung adenocarcinoma samples in TCGA were used (**, p < 0.01). **b** Kaplan-Meier relapse-free survival curves in lung adenocarcinoma patients stratified by GMDS expression level (*p = 0.013*). **c** Quantified GMDS protein level in 75 paired lung adenocarcinoma samples examined by Immunohistochemistry (**, p < 0.01). **d** Representative images of GMDS IHC staining in human lung adenocarcinoma and adjacent normal tissues. (Magnification, left is × 20, right is × 100). **e** GMDS protein level in different cell lines including BEAS-2B (1), MRC-5 (2), HEK-293 (3), A549 (4), H1299 (5), SPC-A-1 (6). A59, H1299 and SPC-A-1 are all human lung adenocarcinoma cell lines

To identify candidate genes involved in human lung adenocarcinoma tumorigenesis, transcriptomes of 57 paired lung adenocarcinoma tissues were selected from TCGA database and gene profiling analysis were performed. It was shown that GMDS expression at mRNA level was significantly upregulated in lung adenocarcinoma tissues as compared to adjacent normal tissues (Fig. 1a). We then examined the correlation between GMDS expression at mRNA level and prognosis in one patient cohort (using data set GSE31210), and revealed that higher GMDS expression was correlated with poor prognosis of lung adenocarcinoma patients (Fig. 1b). Without fresh specimens in hand, we further examined GMDS expressions using immunohistochemistry with only one suitable antibody with tissue microarray and confirmed the upregulation of GMDS expression at protein level in human lung adenocarcinoma, with GMDS protein density at 3.597 ± 1.908 in human lung adenocarcinoma and 0.453 ± 1.119 in adjacent normal tissues (Fig. 1c-d). Then relationship between GMDS protein level and clinicopathological parameters was analyzed. However, no obvious correlation was observed between GMDS and any clinicopathological parameters including gender, age, tumor size and pathologic grades (Table 1). In addition, GMDS protein expression in common lung adenocarcinoma cell lines A549, H1299 and SPC-A-1 was examined. As compared to normal cell lines BEAS-2B, MRC-5 and HEK-293 cells, GMDS protein was significantly upregulated in A549 and H1299 cells (Fig. 1e), both of which were used for subsequent functional analysis. It is possible that GMDS might be involved in the early stage of lung adenocarcinoma development, so the impact of GMDS expression on cell proliferation and survival in lung adenocarcinoma was examined in the following studies.

### Silencing of GMDS expression in human lung adenocarcinoma cells with lentiviral-mediated shRNA strategy

**Fig. 2:**
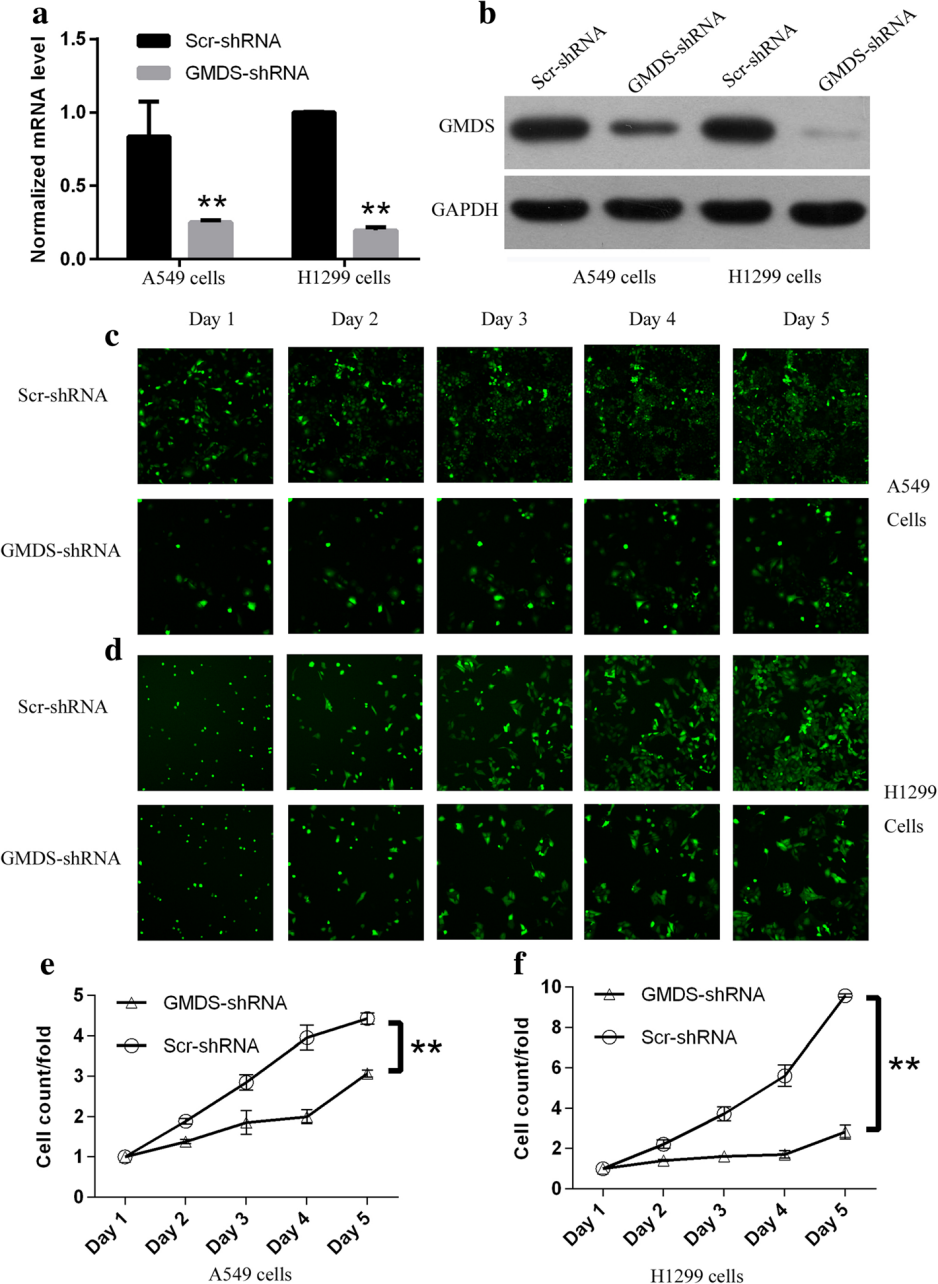
Impaired cell proliferation in human lung adenocarcinoma cell lines with GMDS knockdown via Cellomics ArrayScan VTI. **a** GMDS mRNA level in A549 cells and H1299 cells infected with lentivirus expressing either Scr-shRNA or GMDS-shRNA examined by quantitative real-time PCR (normalized to GAPDH mRNA). Data shown here was one out of three independent experiments (**, p < 0.01). **b** Relative GMDS protein level in A549 cells and H1299 cells infected with lentivirus expressing either Scr-shRNA or GMDS-shRNA examined by western blot. GAPDH protein was used as internal control. c-d. Representative microscope pictures of A549 cells (**c**) and H1299 cells (**d**) infected with lentivirus expressing either Scr-shRNA or GMDS-shRNA at different time points. e-f. Proliferation profiling of A549 cells (**e**) and H1299 cells (**f**) infected with lentivirus expressing either Scr-shRNA or GMDS-shRNA for continuous 5 days examined by Cellomics ArrayScan VTI. Histogram shown here was relative fold changes of cell numbers compared to Day 1 and representing the mean ± SEM of three independent experiments (**, p < 0.01)

To inhibit GMDS expression in human lung adenocarcinoma cells efficiently, RNA interference (RNAi) based on lentivirus was used for GMDS knockdown in two human lung adenocarcinoma cells A549 and H1299. A549 cells and H1299 cells were infected with lentivirus expressing either scrambled shRNA (Scr-shRNA) or human GMDS-specific shRNA (GMDS-shRNA). Lentivirus used here contained GFP expression cassette, which served as labeling marker for infection efficiency and cells with infection efficiency > 80% were used for subsequent functional analysis. Knockdown efficiency of GMDS shRNA was examined using quantitative real-time PCR. It was shown that approximately 70 and 80% reduction of GMDS expression at mRNA level in A549 and H1299 cell lines infected with lentivirus expressing GMDS-shRNA as compared to control group, respectively (Fig. 2a). Furthermore, knockdown efficiency of GMDS shRNA at protein level was determined in both A549 and H1299 cell lines using western blot assay and GMDS protein level reduced significantly in cells infected with lentivirus expressing GMDS-shRNA as compared to control group (Fig. 2b).

### Impairment of cell proliferation ability by GMDS knockdown in human lung adenocarcinoma cells

Two human lung adenocarcinoma cell lines A549 and H1299 were infected with lentivirus expressing either Scr-shRNA or GMDS-shRNA to investigate the impact of GMDS on cell growth of lung adenocarcinoma. After culturing for 24 h, cell numbers were quantified using the high-content screening system Cellomics ArrayScan VTI every day for continuous 5 days (Representative images for A549 and H1299 cells shown in Fig. 2c-d). Results showed that impaired cell proliferation was obvious in both cell lines at Day 2 and the inhibitory impact of GMDS knockdown on cell proliferation was more obvious in the following 3 days (Fig. 2e-f).

**Fig. 3:**
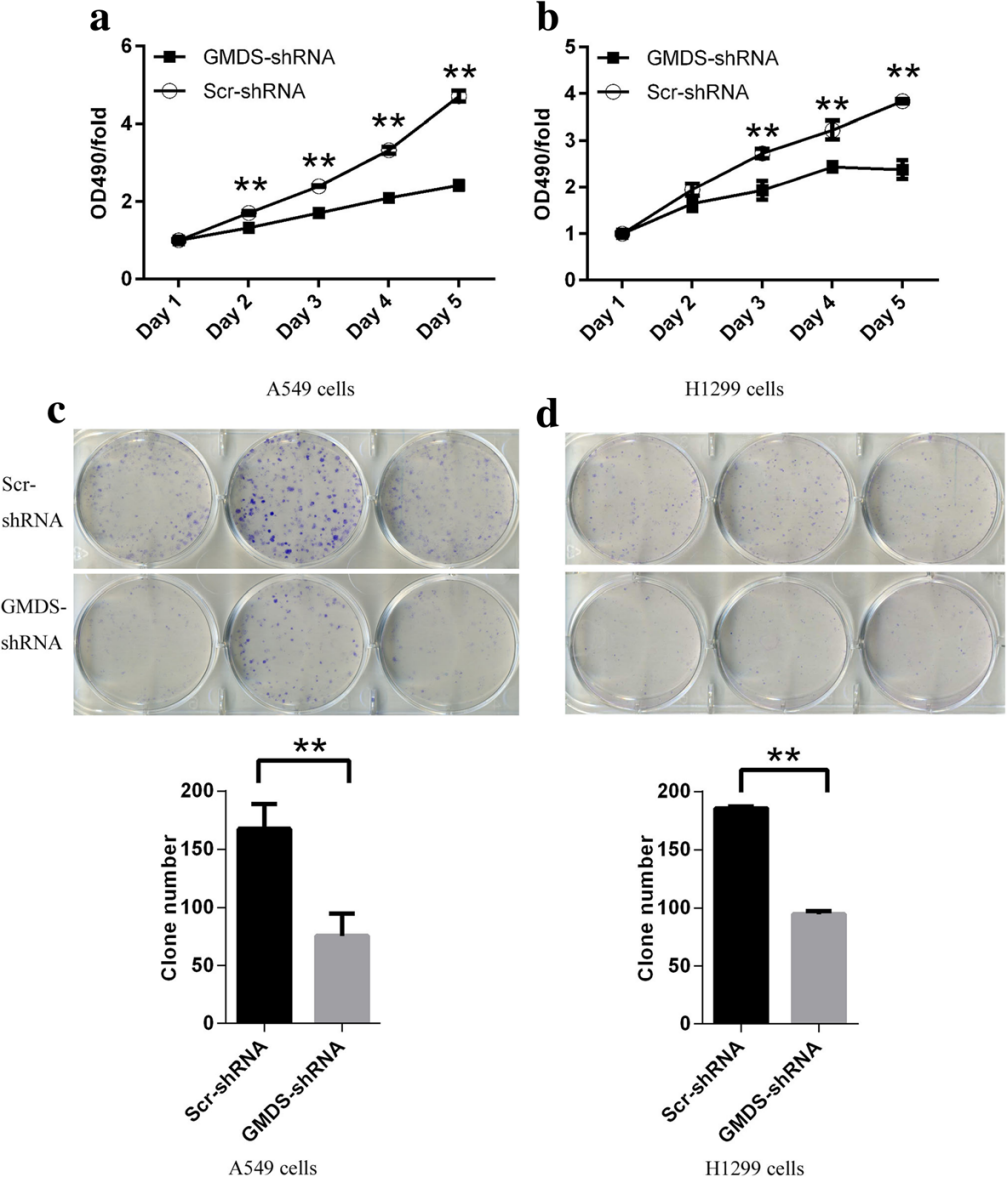
Impaired cell proliferation colony formation in human lung adenocarcinoma cell lines with GMDS knockdown. **a**-**b** Cell proliferation profiling in A549 cells (**a**) and H1299 cells (**b**) infected with lentivirus expressing either Scr-shRNA or GMDS-shRNA for continuous 5 days analyzed by MTT assay. Data shown here was relative fold changes of absorbance at OD490 compared to Day 1 and representing the mean ± SEM of three independent experiments (**, p < 0.01). **c**-**d** Colony formation in A549 cells (**c**) and H1299 cells (**d**) infected with lentivirus expressing either Scr-shRNA or GMDS-shRNA. Histogram shown here was average colony numbers and representing the mean ± SEM of three independent experiments (**, p < 0.01)

As unlimited cell proliferation ability was the key feature of tumor initiation and development, we further determined the influence of GMDS knockdown on cell proliferation ability in both lung adenocarcinoma cell lines A549 and H1299 with MTT assay for continuous 5 days. In consistent with results obtained with Cellomics ArrayScan VTI assay, MTT assay also revealed that GMDS knockdown led to impaired cell proliferation ability in both cell lines (Fig. 3a-b). In A549 cells, impaired cell proliferation was observed at Day 2 and the inhibitory effect was more obvious in the following 3 days (Fig. 3a) while in H1299 cells, impaired cell proliferation was observed at Day 3 and the inhibitory effect was still obvious in the following 2 days (Fig. 3b). Taken together, both Cellomics ArrayScan VTI and MTT assay confirmed that GMDS knockdown led to impaired cell proliferation ability in A549 and H1299 cell lines.

### Inhibition of colony formation by GMDS knockdown in human lung adenocarcinoma cells

Colony formation assay was used here to examine the impact of GMDS knockdown on colony formation ability in human lung adenocarcinoma cells A549 and H1299. After culturing for 14 days, the colony formation ability was inhibited significantly in both A549 and H1299 cells infected with GMDS-shRNA as compared to cells infected with Scr-shRNA (Fig. 3c-d). The average colony number reduced to 75 from 167 in A549 cells while the average number reduced to 95 from 185 in H1299 cells.

### Cell cycle arrest induced by GMDS knockdown in human lung adenocarcinoma cells

**Fig. 4:**
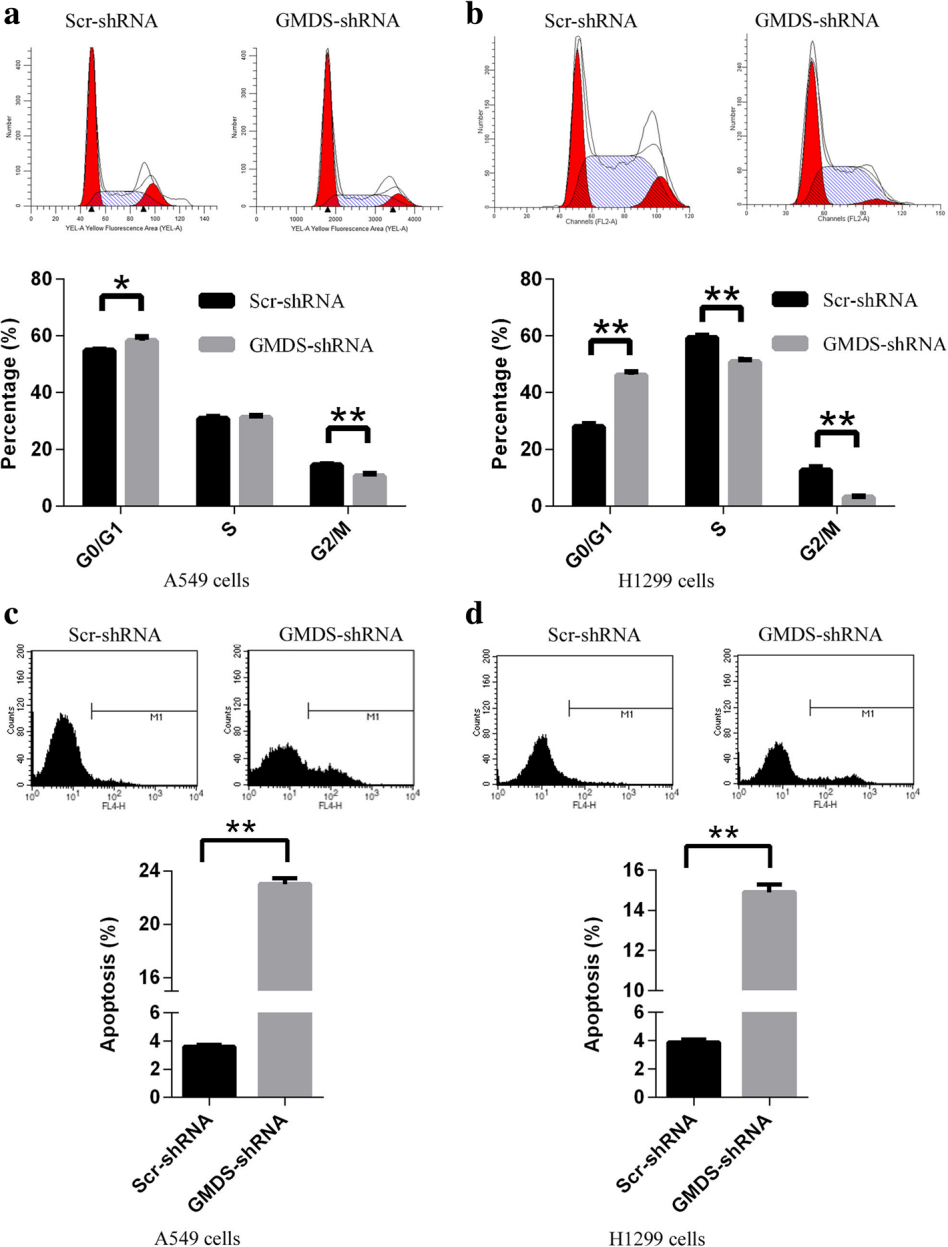
Abnormal Cell cycle process and cell apoptosis in human lung adenocarcinoma cell lines with GMDS knockdown. **a-b** Cell cycle distribution in A549 cells (**a**) and H1299 cells (**b**) infected with lentivirus expressing either Scr-shRNA or GMDS-shRNA. Both cells infected with lentivirus expressing either Scr-shRNA or GMDS-shRNA were cultured for 72 h. After propidium iodide (PI) staining, cell cycle distribution was analyzed with flow cytometry. The graph represents the mean ± SEM of cell proportion in the G1 phase, S phase and G2/M phase from three independent experiments (*, p < 0.05; **, p < 0.01). **c**-**d** Cell apoptosis in A549 cells (**c**) and H1299 cells (**d**) infected with lentivirus expressing either Scr-shRNA or GMDS-shRNA. Both cells infected with lentivirus expressing either Scr-shRNA or GMDS-shRNA were cultured for 4 days and cell apoptosis was analyzed using ANNEXIN-V assay in combined with flow cytometry. Data shown are the mean ± SEM of cell percentage in apoptosis from three independent experiments (**, p < 0.01)

Cell proliferation and colony formation ability was closely associated with cell cycle progression, so it is necessary to examine the influence of GMDS knockdown on cell cycle process to elucidate underlying mechanisms for GMDS-mediated cell growth abnormalities. Human lung adenocarcinoma cell lines A549 and H1299 were infected with lentivirus expressing either Scr-shRNA or GMDS-shRNA and cell cycle distribution was analyzed using propidium iodide (PI) staining in combined with fluorescence-activated cell sorting (FACS). It was shown that cell cycle arrest was induced by GMDS knockdown in both cell lines at 72 h after lentiviral infection. In A549 cells, GMDS knockdown led to a significant G1 arrest with a parallel G2/M-phase reduction and cell percentage at G0/G1 phase, S phase and G2/M phase in cells infected with GMDS-shRNA as compared to Scr-shRNA was 55.8% vs 58.2, 30.7% vs 31.1, and 14.4% vs 10.6%, respectively (Fig. 4a); while in H1299 cells, GMDS knockdown led to a significant G1 arrest with a parallel S and G2/M-phase reduction and cell percentage at G0/G1 phase, S phase and G2/M phase in cells infected with GMDS-shRNA as compared to Scr-shRNA was 28% vs 46.1, 59.4% vs 50.8, and 12.6% vs 3.1%, respectively (Fig. 4b). Furthermore, similar cell cycle arrest was also induced by GMDS knockdown in both cell lines at 48 h after lentiviral infection (Additional file 1: Figure S1a-b). These results implies that GMDS participates in cell cycle regulation in human lung adenocarcinoma.

### Induction of cell apoptosis by GMDS knockdown in human lung adenocarcinoma cells

Evasion of apoptosis is another hallmark of cancer, and would also promote cell growth and proliferation, so it is important to examine the cell apoptotic status in human lung adenocarcinoma cells infected with lentivirus expressing GMDS-shRNA. Cell apoptosis was analyzed using annexin V-APC assay in combined with FACS technology in A549 and H1299 cells infected with lentivirus expressing either Scr-shRNA or GMDS-shRNA after culturing for 4 days. In A549 and H1299 cells infected with lentivirus expressing Scr-shRNA, apoptosis was observed in about 4% cells while in cells infected with lentivirus expressing GMDS-shRNA, percentage of cell apoptosis reached ~ 23% in A549 cells and ~ 15% in H1299 cells (Fig. 4c-d).

### In vivo tumorigenicity was impaired in the xenograft mice model of lung adenocarcinoma by GMDS knockdown

**Fig. 5:**
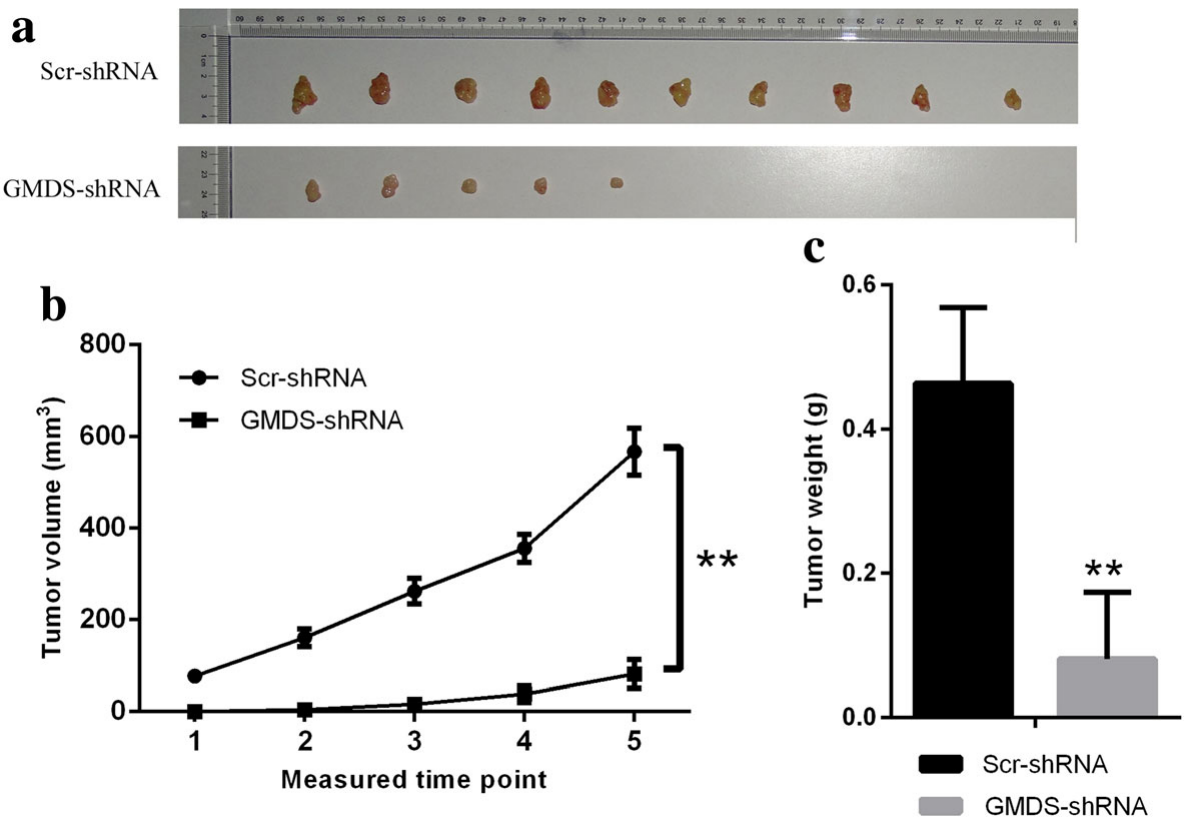
Tumorigenesis was inhibited by GMDS knockdown in xenograft mice model of lung adenocarcinoma. **a** Representative tumor pictures from nude mice injected subcutaneously with lung adenocarcinoma cell line H1299 cells infected with either Scr-shRNA or GMDS-shRNA. Tumor volume was examined from the 10th day after H1299 cell injections for 5 times with a frequency of 2 times a week. Nude mice were killed at the 29th day to check tumor weight. **b** Tumor volume in nude mice injected subcutaneously with lung adenocarcinoma cell line H1299 cells infected with either Scr-shRNA or GMDS-shRNA. Data shown here is the mean ± SEM of tumor volume from 10 nude mice in each group (**, p < 0.01). **c** Tumor weight in nude mice injected subcutaneously with lung adenocarcinoma cell line H1299 cells infected with either Scr-shRNA or GMDS-shRNA. Data shown are the mean ± SEM of tumor weight from 10 nude mice in each group (**, p < 0.01)

Our studies in two human lung adenocarcinoma cells A549 and H1299 cells confirmed that GMDS was important for tumorigenesis of lung adenocarcinoma in vitro. However, in vivo evidence for the involvement of GMDS in tumorigenesis of lung adenocarcinoma remained elusive. Here we established xenograft mice model of lung adenocarcinoma with subcutaneously injection of H1299 cells infected with lentivirus expressing either Scr-shRNA or GMDS-shRNA into nude mice. These mice were fed for 29 days for tumor volume and weight analysis. As shown in Fig. 5a, Reduction of tumor size was obvious in nude mice injected with cells infected with lentivirus expressing GMDS-shRNA as compared to cells infected with lentivirus expressing Scr-shRNA. Then the tumor volume was quantified and confirmed that GMDS knockdown inhibited tumor growth at all 5 measured time points (Fig. 5b). What’s more, tumor weight was significantly lower in nude mice injected with GMDS-shRNA cells (Fig. 5c) as compared to nude mice injected with Scr-shRNA cells, as the mean weight was reduced from 0.46 g to 0.08 g.

### Global gene expression changes in human lung adenocarcinoma cells with GMDS knockdown

**Fig. 6:**
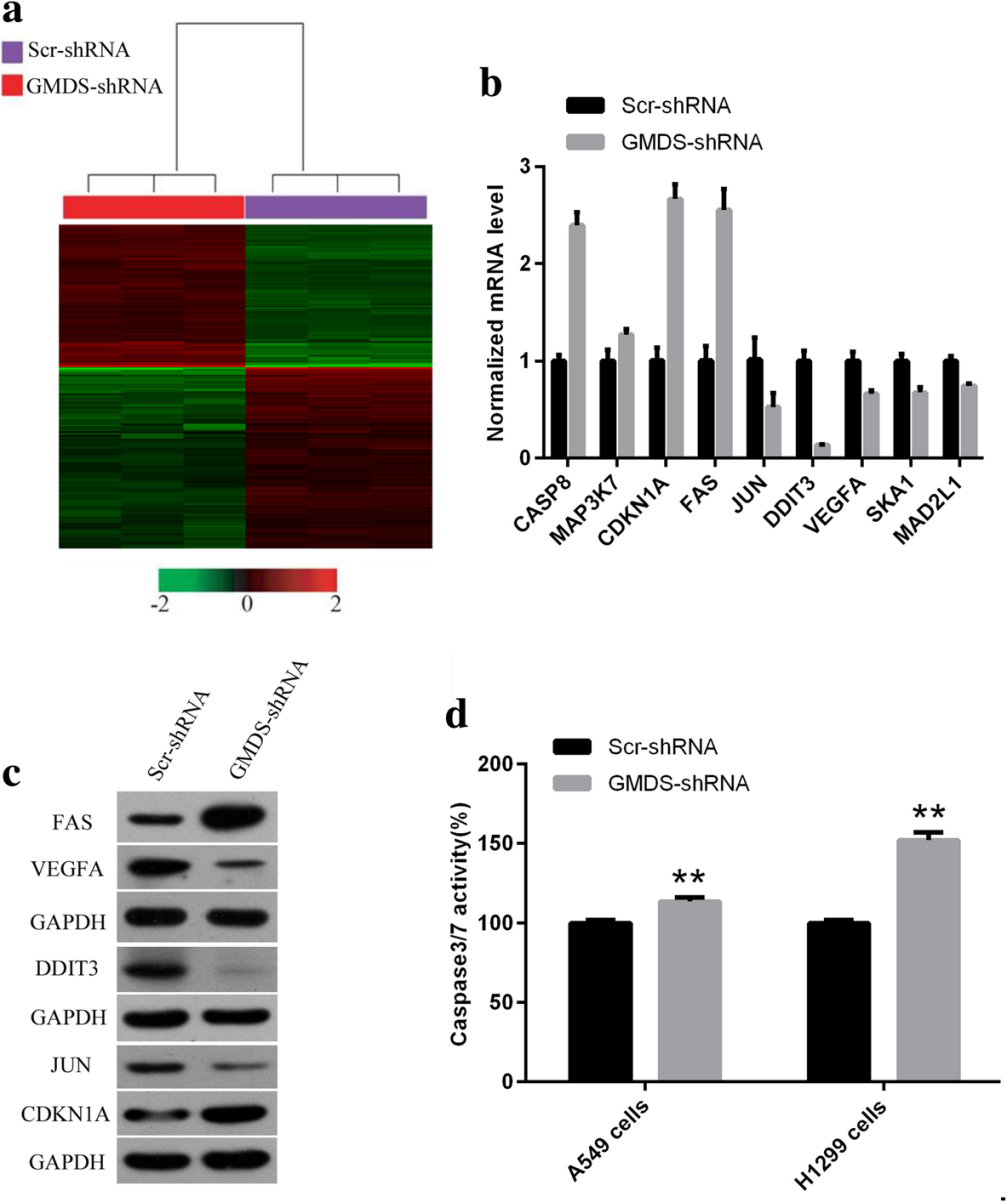
Widespread changes of gene expressions and critical pathways in human lung adenocarcinoma cells A549 with GMDS knockdown. **a** Heatmap containing 739 differentially expressed genes in human lung adenocarcinoma cell line A549 infected with lentivirus expressing either Scr-shRNA (purple) or GMDS-shRNA (red) with the criteria *P* < 0.05 and ▏fold change ▏ > 1.5. Genes and samples were listed in rows and columns, respectively. A colour standard for normalized expression data was shown at the bottom of the microarray heatmap (green represents downregulated genes while red represents upregulated genes). **b** Gene expression changes identified in microarray were confirmed using real-time quantitative PCR for selected genes CASP8, MAP3K7, CDKN1A, FAS, JUN, DDIT3, VEGFA, SKA1 and MAD2L1 in human lung adenocarcinoma cell line A549 infected with lentivirus expressing either Scr-shRNA or GMDS-shRNA. Histogram shown here was one out of three independent experiments (*p < 0.01*) and normalized to GAPDH. **c** Protein level of FAS, VEGFA, DDIT3, JUN and CDKN1A in human human lung adenocarcinoma cell line A549 infected with lentivirus expressing either Scr-shRNA or GMDS-shRNA. GAPDH was used as internal control. **d** Caspase3/7 activity analysis in A549 cells and H1299 cells infected with lentivirus expressing either Scr-shRNA or GMDS-shRNA. Data shown are the mean ± SEM of caspase3/7 activity from three independent experiments (**, p < 0.01)

**Fig. 7:**
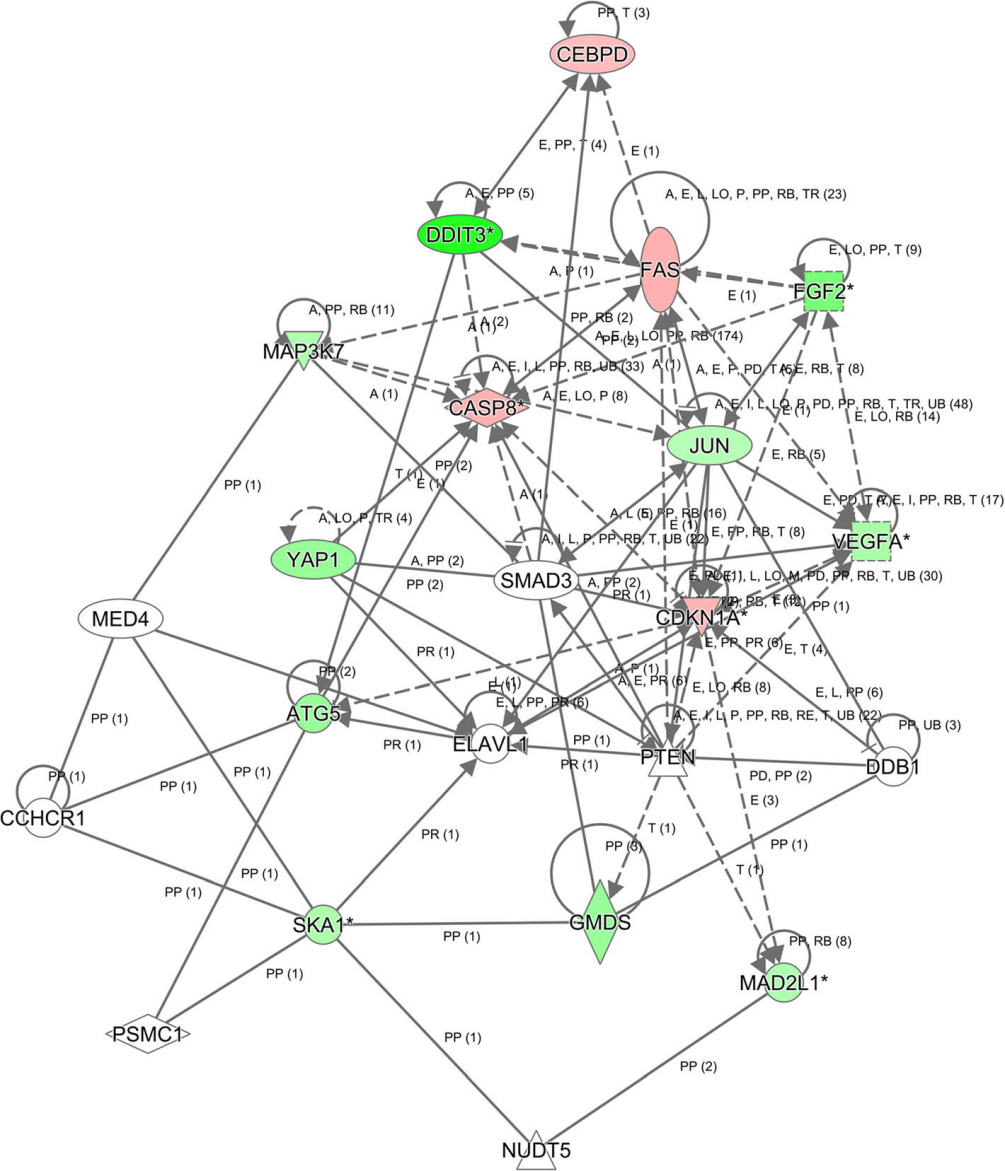
Modulation of CDKN1A-CDK1-mediated gene interaction network in human lung adenocarcinoma cell line A5492 cells after GMDS knockdown. Genes represented in green were downregulated while genes in red were upregulated in A549 cells after GMDS knockdown. Solid line represented direct interactions while dotted line represented indirect interactions. The meaning of abbreviations in the figure: A, activation; B, binding; C, causation/leads to; CO, correlation; CC, chemical-chemical interaction; CP, chemical-protein interaction; E, expression; EC, enzyme catalysis; I, inhibition; L, molecular cleavage; LO, localization; M, biochemical modification; miT, microRNA targeting; MB, group/complex membership; nTRR, non-targeting RNA-RNA interaction; P, phosphorylation/dephosphorylation; PD, protein-DNA binding; PP, protein-protein binding; PR, protein-RNA binding; PY, processing yields; RB, regulation of binding; RE, reaction; RR, RNA-RNA binding; T, transcription; TR, translocation; UB, ubiquitination

To elucidate the underlying molecular mechanisms of the tumor suppressive roles of GMDS knockdown in human lung adenocarcinoma cells, microarray analysis was performed to examine the gene expression profiling of A549 cells infected with lentivirus expressing either Scr-shRNA or GMDS-shRNA. A total of 739 genes showing significantly expression changes were detected, with *P* < 0.05 and > 1.5 absolute value of fold change, including 316 upregulated genes and 423 downregulated genes (Fig. 6a). Then, disease and functional analysis with Ingenuity Pathway Analysis (IPA) revealed that cell cycle and cellular growth and proliferation were enriched in GMDS-regulated gene sets in human lung adenocarcinoma cells. Furthermore, upstream analysis with IPA revealed that GMDS knockdown stimulated CDKN1A (p21) and downstream pathways significantly (*P =* 1.1E-07). Then real-time PCR and western blot were performed to examine the impact of GMDS knockdown on the status of CDKN1A-associated pathways including factors CDKN1A, CASP8, MAP3K7, FAS, JUN, DDIT3, VEGFA, SKA1 and MAD2L1. It was shown that GMDS knockdown induced the expression of CDKN1A, CASP8, MAP3K7 and FAS while inhibited JUN, DDIT3, VEGFA, SKA1 and MAD2L1 at mRNA level (Fig. 6b). Furthermore, at the protein level CDKN1A and FAS were also enhanced while VEGFA, DDIT3 and JUN were inhibited by GMDS knockdown (Fig. 6c). Out of these GMDS-regulated genes, it was noticed that cell death and survival was the most affected biological process, so we further performed caspase3/7 analysis to examine the impact of GMDS knockdown on caspase-mediated cell apoptosis process. It was shown that GMDS knockdown enhanced caspase3/7 activity significantly in both A549 cells and H1299 cells (Fig. 6d). Furthermore, IPA network analysis showed that CASP8-CDKN1A axis was at the core of GMDS-associated gene interaction network, emphasizing the core of CDKN1A in GMDS-mediated lung adenocarcinoma growth (Fig. 7).

## Discussion

Glycosylation is an important post-translational modification occurred in more than half of all known human proteins and can be classified into N-glycans or O-glycans depending on the covalently attachment patterns of glycans via either nitrogen or oxygen linkages, respectively [11]. In terms of the complexity, glycosylation exceeds most of other PTMs as it involves the linkage of divergent carbohydrates ranging from monosaccharide to oligosaccharides and could occur in at least 9 of the 20 amino acids, so it is not surprising that glycosylation was essential for many biological processes and its abnormalities account for many human diseases including cancer [10, 23]. Indeed, glycosylation alterations in tumor cells influence cell growth and survival, tumor cell invasion and metastasis, tumor angiogenesis and cell-microenvironment interactions [24–26]. In lung cancer and especially lung adenocarcinoma, the involvement of glycosylation abnormalities in tumorigenesis has been established previously [27, 28]. However, studies on genes responsible for glycosylation dysfunctions in lung adenocarcinoma are still limited. Here we focused on GMDS, a gene involved in glycosylation, and confirmed its tumor-promoting role in lung adenocarcinoma in vitro and in vivo.

GMDS is an important enzyme involved in guanosine diphosphate (GDP)-fucose synthesis and GDP-fucose is the donor substrate of fucosylation, one of the most common type of cancer-associated glycosylation alterations [29]. Fucosylation abnormalities have been observed in many cancer types including colorectal cancer, hepatocellular carcinoma and papillary carcinoma of the thyroid [15, 30–32]. As a critical enzyme in fucosylation, GMDS deregulation was also detected in colorectal cancer and GMDS dysfunction led to tumor escape and resistance to cellular apoptosis in colorectal cancer cells [16–18]. However, these studies were just performed at cell level and the expression status of GMDS in clinical caner specimens has not been examined. What’s more, roles of GMDS in lung adenocarcinoma have not been described previously, so according to our knowledge, this study was the first to systematically analyze the functional impact and molecular mechanisms of GMDS in lung adenocarcinoma in vitro and in vivo. We first examined GMDS expression at mRNA level in human lung adenocarcinoma using transcriptome data of 57 paired human lung adenocarcinoma tissues from TCGA database and showed that GMDS was upregulated in human lung adenocarcinoma as compared to adjacent normal tissue. We further examined GMDS protein density using tissue microarray in paired human lung adenocarcinoma samples and confirmed the upregulation of GMDS in human lung adenocarcinoma. However, no significant correlation was observed between GMDS expression and any clinical pathological parameters, which suggests that GMDS might be involved in the early stage of lung adenocarcinoma development. Functional importance of GMDS in tumorigenesis was confirmed as it was shown that GMDS knockdown led to delayed cell proliferation, impaired colony formation ability, cell cycle arrest and increased apoptosis. Xenograft tumor mouse model experiments further revealed that GMDS knockdown inhibited tumor growth in vivo. Taken together, these results confirmed that GMDS is involved in tumorigenesis of lung adenocarcinoma.

In accord with the tumor-promoting roles of GMDS in lung adenocarcinoma described above, gene expression profiling analysis with microarray showed that genes essentially for cell survival and proliferation were regulated by GMDS. We revealed that CDKN1A (p21) and downstream pathways were activated in cells treated with GMDS-shRNA by IPA analysis. Further network analysis revealed that CASP8-CDKN1A axis was at the core of GMDS-mediated gene expression dataset. Both confirmed that CDKN1A was the core of GMDS-mediated lung adenocarcinoma progression. It has been reported that CDKN1A was involved in G1 phase of cell cycle process, cell proliferation and apoptosis [33, 34], which is in accordance with the roles of GMDS in cell proliferation, apoptosis and cell cycle, as GMDS knockdown in lung adenocarcinoma cells led to impaired cell proliferation, enhanced cellular apoptosis and cell cycle retardation at G1 phase revealed in this study. Indeed, GMDS knockdown induced the expression of CASP8 and CDKN1A, which might be the underlying molecular mechanisms for the observation that GMDS knockdown induced cell cycle arrest and cellular apoptosis.

## Conclusions

Taken together, this study provided valuable insights into the molecular mechanisms underlying GMDS-regulated cell growth in lung adenocarcinoma. Microarray and subsequent network analysis are instructive for functional analysis of GMDS in different types of cancer. GMDS upregulation in lung adenocarcinoma implies a potential biomarker and targets for lung adenocarcinoma diagnosis and treatment. In the future, establishment of GMDS-CDKN1A axis and functional investigations would provide more valuable insights into lung adenocarcinoma.

## Additional file

Additional file 1: **Figure S1.** Cell cycle arrest in human lung adenocarcinoma cell lines with GMDS knockdown 48 h after lentiviral infection. a-b. Cell cycle distribution in A549 cells (a) and H1299 cells (b) infected with lentivirus expressing either Scr-shRNA or GMDS-shRNA. Both cells infected with lentivirus expressing either Scr-shRNA or GMDS-shRNA were cultured for 48 h. After propidium iodide (PI) staining, cell cycle distribution was analyzed with flow cytometry. The graph represents the mean ± SEM of cell proportion in the G1 phase, S phase and G2/M phase from three independent experiments (*, p < 0.05; **, p < 0.01). (DOCX 151 kb)

## Abbreviations

FACS: Fluorescence-activated cell sorting
FX: GDP-4-keto-6-deoxymannose-3,5-epimerase-4-reductase
GMDS: GDP-mannose-4,6-dehydratase
NSCLC: Non-small cell lung cancer
PI: Propidium iodide
RNAi: RNA interference
SCLC: Small cell lung cancer
shRNA: Short hairpin RNA
TCGA: The Cancer Genome Atlas
