# Supplementary material for: GMDS knockdown impairs cell proliferation and survival in human lung adenocarcinoma
Source: BMC Cancer. 2018 May 29;18:600. doi: 10.1186/s12885-018-4524-1 (PMC5975429; doi:10.1186/s12885-018-4524-1)
Supplement: Supplementary file 1 — Figure S1. Cell cycle arrest in human lung adenocarcinoma cell lines with GMDS knockdown 48 h after lentiviral infection. a-b. Cell cycle distribution in A549 cells (a) and H1299 cells (b) infected with lentivirus expressing either Scr-shRNA or GMDS-shRNA. Both cells infected with lentivirus expressing either Scr-shRNA or GMDS-shRNA were cultured for 48 h. After propidium iodide (PI) staining, cell cycle distribution was analyzed with flow cytometry. The graph represents the mean ± SEM of cell proportion in the G1 phase, S phase and G2/M phase from three independent experiments (*, p < 0.05; **, p < 0.01). (DOCX 151 kb) [file 12885_2018_4524_MOESM1_ESM.docx]

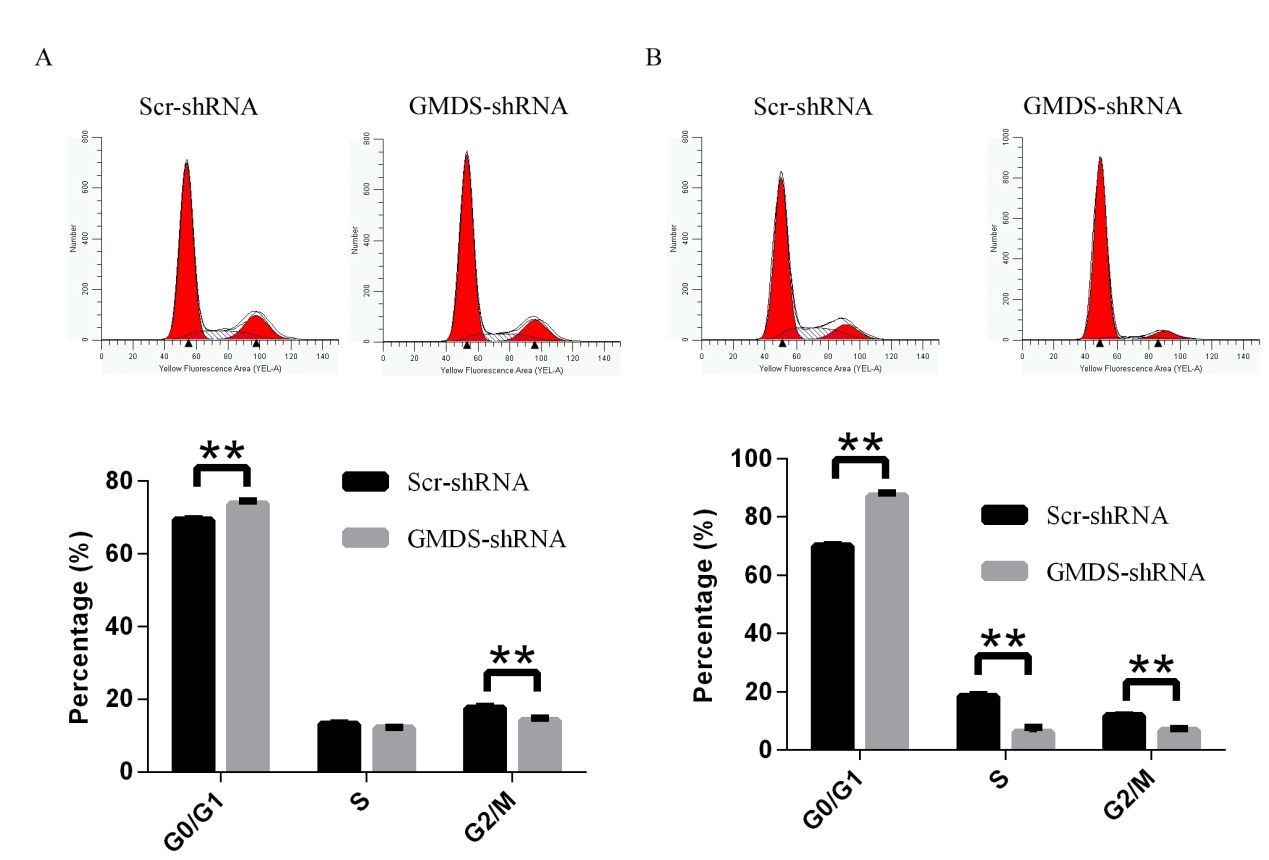


**Fig. S1 Cell cycle arrest in human lung adenocarcinoma cell lines with GMDS knockdown 48 hours after lentiviral infection**

a-b. Cell cycle distribution in A549 cells (a) and H1299 cells (b) infected with lentivirus expressing either Scr-shRNA or GMDS-shRNA. Both cells infected with lentivirus expressing either Scr-shRNA or GMDS-shRNA were cultured for 48 hours. After propidium iodide (PI) staining, cell cycle distribution was analyzed with flow cytometry. The graph represents the mean ± SEM of cell proportion in the G1 phase, S phase and G2/M phase from three independent experiments (**, p<0.05; **, p<0.01*).
